# Supplementary material for: Homotypic SLAMF1:SLAMF1 interactions between innate T cells and neutrophils activate fungal killing by neutrophils
Source: bioRxiv. 2026 Feb 20:2026.02.19.706741. Preprint. [Version 1] doi: 10.64898/2026.02.19.706741 (PMC12934946; doi:10.64898/2026.02.19.706741)

Supplementary Figure 1 to Figure 1

bioRxiv preprint doi: https://doi.org/10.64898/2026.02.19.706741; this version posted February 20, 2026. The copyright holder for this preprint (which was not certified by peer review) is the author/funder, who has granted bioRxiv a license to display the preprint in perpetuity. It is made available under aCC-BY 4.0 International license.

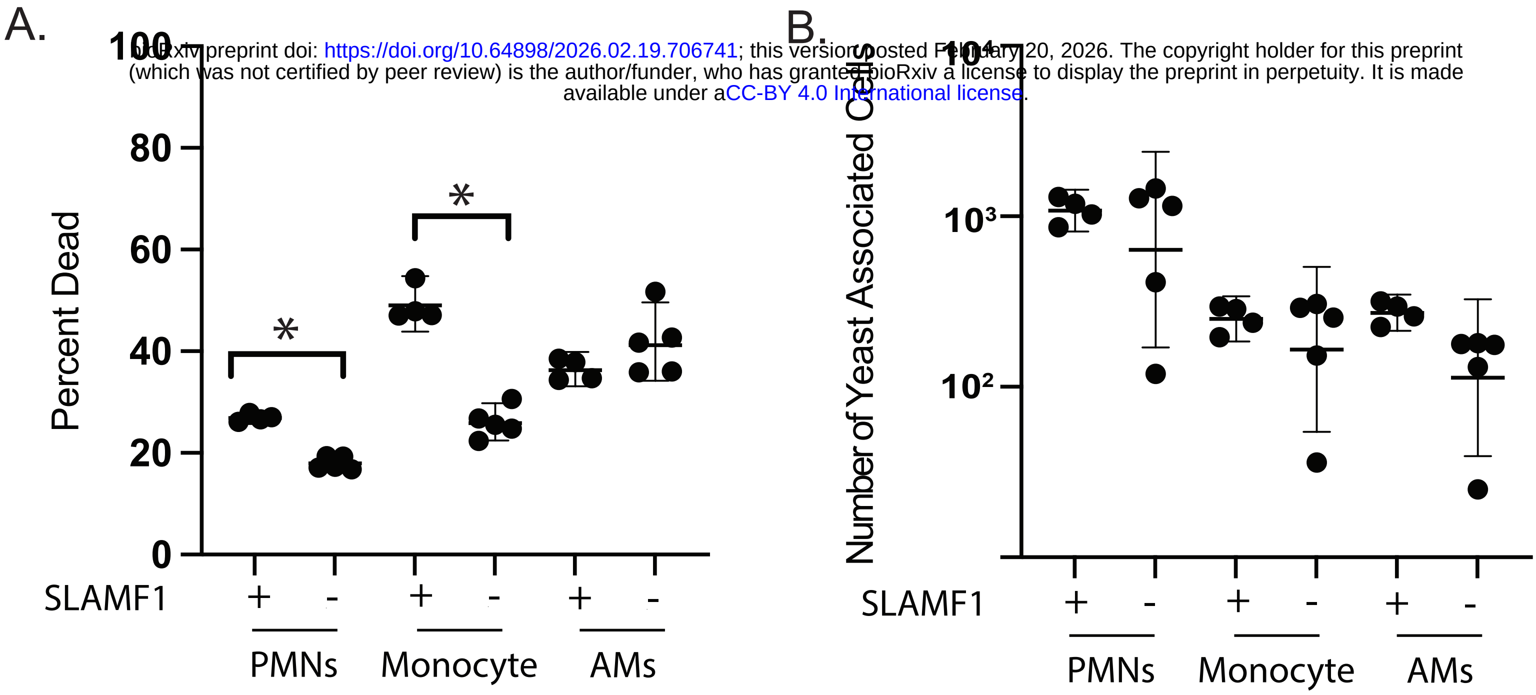

Supplementary Figure 2 to Figure 3

bioRxiv preprint doi: https://doi.org/10.64898/2026.02.19.706741; this version posted February 20, 2026. The copyright holder for this preprint (which was not certified by peer review) is the author/funder, who has granted bioRxiv a license to display the preprint in perpetuity. It is made available under aCC-BY 4.0 International license.

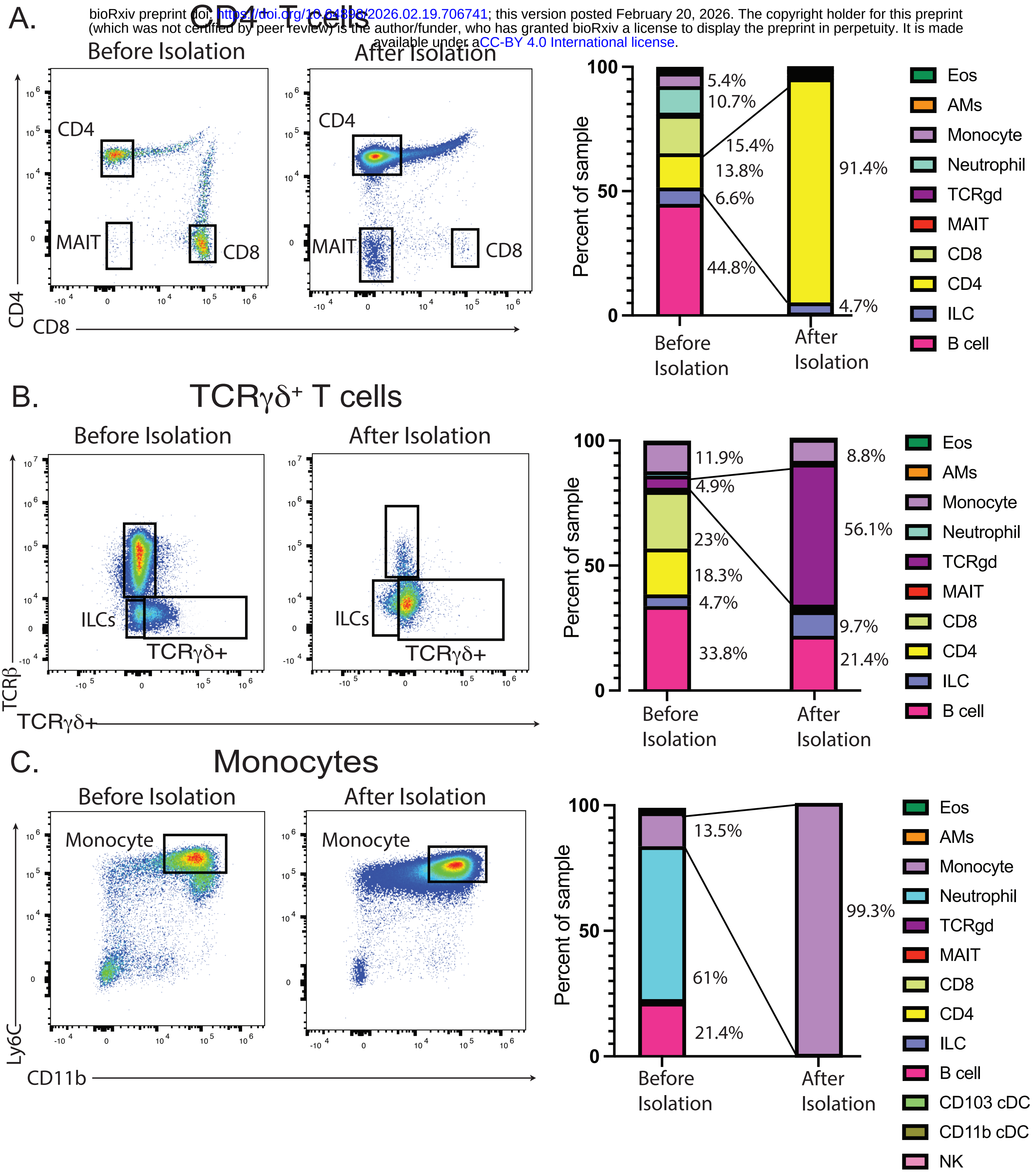

Supplementary Figure 3 to Figure 4

bioRxiv preprint doi: https://doi.org/10.64898/2026.02.19.706741; this version posted February 20, 2026. The copyright holder for this preprint (which was not certified by peer review) is the author/funder, who has granted bioRxiv a license to display the preprint in perpetuity. It is made available under aCC-BY 4.0 International license.

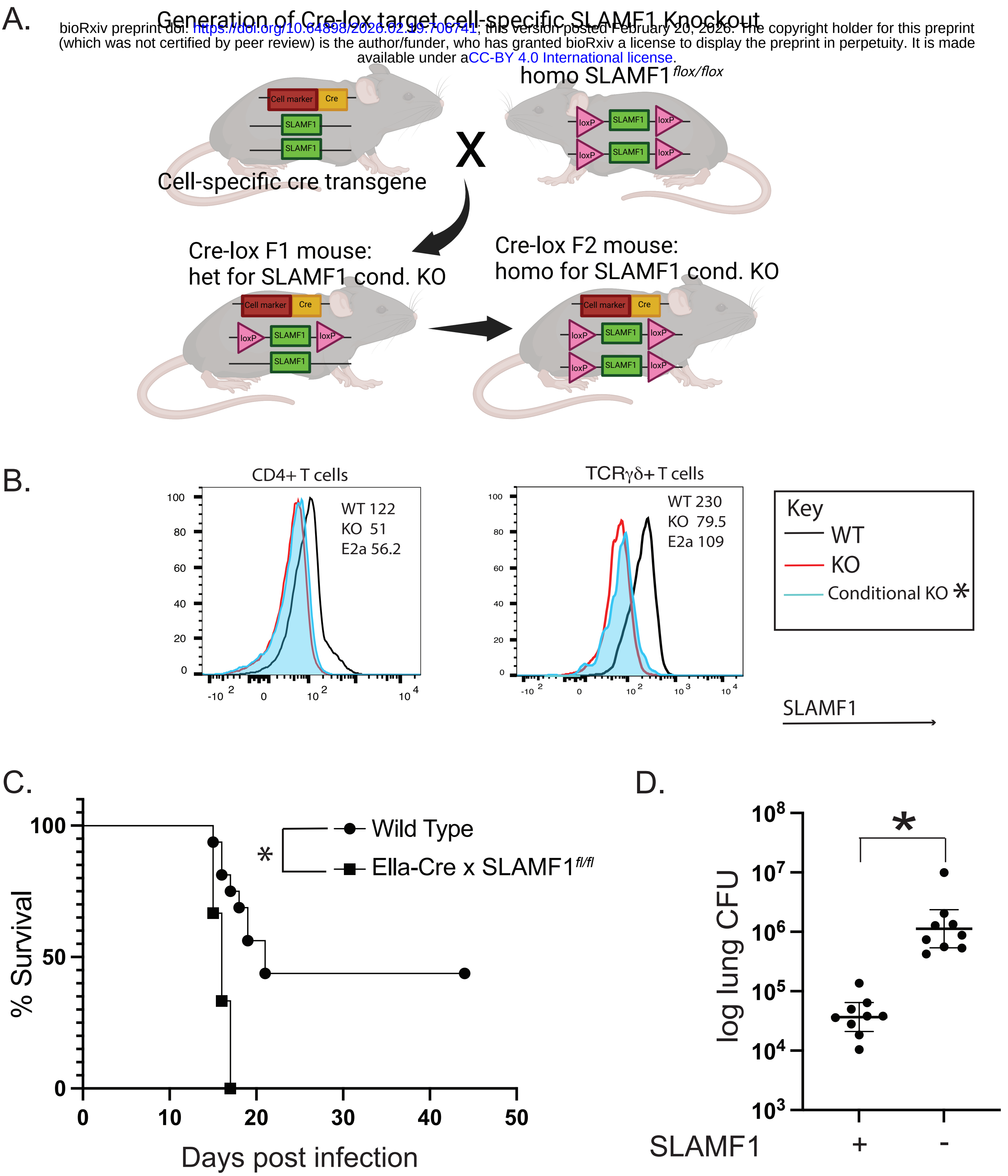

bioRxiv preprint doi: https://doi.org/10.64898/2026.02.19.706741; this version posted February 20, 2026. The copyright holder for this preprint (which was not certified by peer review) is the author/funder, who has granted bioRxiv a license to display the preprint in perpetuity. It is made available under aCC-BY 4.0 International license.

Supplementary Figure 4 to Figure 4

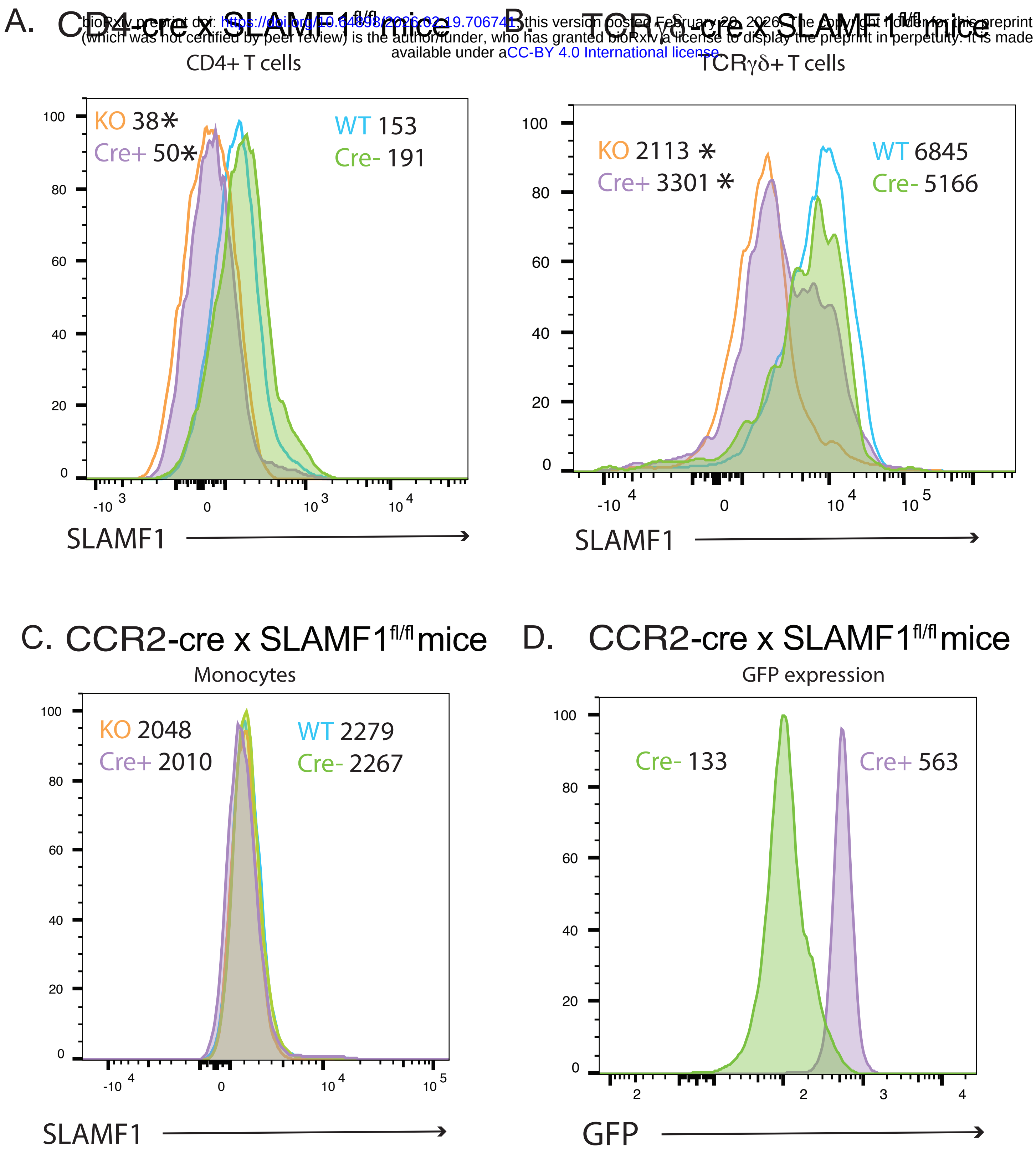

Supplement: Supplement 1 — Supplementary Figure 1 to main Figure 1 In vivo killing assay: Percent of lung phagocytes associated with dead yeast (A) and number of alveolar macrophages, neutrophils, and monocytes associated with total number of yeast for wild type and SLAMF1 knockout mice (B). *p<0.05, two tailed Mann-Whitney T test. Supplementary Figure 2 to main Figure 3 Flow analysis and stacked bar graphs of lung leukocyte populations before and after isolation of CD4+ T cells (A) and TCRγδ+ T cells (B), and analysis of bone marrow before and after isolation of monocytes (C). To identify cell types of interest before and after enrichment we used a panel of multiplexing antibodies to identify 17 pulmonary leukocyte subsets with 12 color flow cytometry [16]. Stacked bar graphs show percentage of each immune cell type relative to all immune cells identified by the Lymphoid Myeloid panel. Supplementary Figure 3 to main Figure 4 Breeding scheme for the generation of conditional SLAMF1knockout mice. To validate commercial SLAMF1 floxed mice we bred them first to homozygosity and crossed them with Ella-Cre mice to generate Ella-cre x SLAMF1fl/fl mice that lack SLAMF1 in embryonic cells (A). SLAMF1 expression on target cells for Ella-cre x SLAMF1fl/fl mice in comparison to wildtype and SLAMF1 KO mice. Geometric MFI of staining. Plots are concatenates from 5 mice/group. *p<0.05 vs WT, two tailed Mann-Whitney T test (B). Survival (C) and lung CFU (D) 9 days post infection of Ella-cre x SLAMF1fl/fl mice and WT mice infected with Bd. *p<0.05, Kaplan Meier test for survival. CFU from at least 9 mice/group are expressed as Log10 plotted with geometric mean ± geometric SD *p<0.05 two tailed Mann-Whitney T test. Supplementary Figure 4 to Figure 4 Ex vivo staining of lung leukocytes for SLAMF1 in CD4-cre x SLAMF1fl/fl mice (A) and TCRγδ-cre x SLAMF1fl/fl mice (B). Ex vivo staining of bone marrow for SLAMF1 in CCR2-cre x SLAMF1fl/fl mice (C). Plots show the histograms and geometric mean fluorescent intensity [file NIHPP2026.02.19.706741v1-supplement-1.pdf]
